# Tetraphenylethene-Modified Colorimetric and Fluorescent Chemosensor for Hg^2+^ With Aggregation-Induced Emission Enhancement, Solvatochromic, and Mechanochromic Fluorescence Features

**DOI:** 10.3389/fchem.2021.811294

**Published:** 2022-01-26

**Authors:** Jin-jin Tian, Dian-dian Deng, Long Wang, Zhao Chen, Shouzhi Pu

**Affiliations:** ^1^ Jiangxi Key Laboratory of Organic Chemistry, Jiangxi Science and Technology Normal University, Nanchang, China; ^2^ Department of Ecology and Environment, Yuzhang Normal University, Nanchang, China

**Keywords:** rhodanine, chemosensor, aggregation-induced emission enhancement, solvatochromic fluorescence, mechanochromic fluorescence

## Abstract

A tetraphenylethene (TPE)-modified rhodanine derivative was successfully designed and prepared, and this luminophor showed intramolecular charge transfer nature from the TPE unit to the rhodanine-3-acetic acid unit. Interestingly, this luminogen not only exhibited typical aggregation-induced emission enhancement (AIEE) behavior but also showed good cell imaging performance. Remarkably, this AIEE-active TPE-containing rhodanine derivative possessed noticeable solvatochromic fluorescence effect involving multiple fluorescent colors of green, yellow-green, yellow, orange, and red. Meanwhile, this fluorescigenic compound displayed reversible mechanochromic fluorescence behavior based on the mutual transformation of between stable crystalline and metastable amorphous states. On the other hand, this multifunctional fluorophor could selectively and sensitively detect Hg^2+^ in an acetonitrile solution. Furthermore, this chemosensor could also be used to detect Hg^2+^ on test paper strips.

**GRAPHICAL ABSTRACT:**
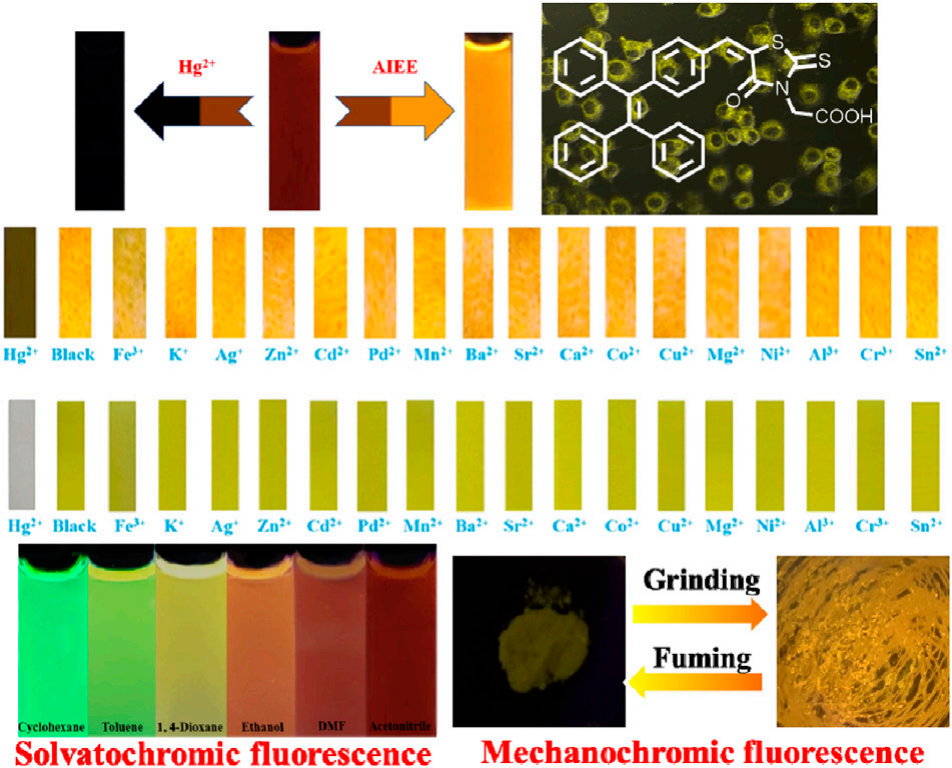
An AIEE-active colorimetric and fluorescent chemosensor of Hg^2+^ was reported, and it exhibited good cell imaging, notable solvatofluorochromic and reversible mechanofluorochromic characteristics.

## Introduction

The exploitation of fluorescent chemosensors for the detection of transition-metal ions has received immense interest because contamination caused by heavy metal ions may have adverse influence on human health and the natural environment (Que et al., 2008; Zhang et al., 2011). Among various transition metal ions, Hg^2+^ is regarded as one of the most dangerous. For example, Hg^2+^ can go through the biofilm and produces serious harm to the central nervous system and endocrine system (Tan et al., 2017; Feng et al., 2017; Gupta et al., 2017; Yang et al., 2018; Li et al., 2018). Indeed, it can induce the formation of many diseases containing kidney failure and cognitive and motion disorder (Bhalla et al., 2010; Yang et al., 2014). Therefore, it is a significant research topic to develop high-efficiency methods for detecting Hg^2+^. Notably, fluorescent sensing has become a powerful tool for the detection of Hg^2+^ (Kiani et al., 2020; Singh et al., 2020; Xu et al., 2020; Tian et al., 2020; Nan et al., 2021). Highly emissive materials in the aggregation state have drawn much attention because of their broad applications in chemical sensing, bioimaging, and photoelectric devices (Tong et al., 2014; Huang et al., 2014; Song et al., 2016; Tang et al., 2019; Zheng et al., 2019; Li et al., 2021; Cheng et al., 2021; Yin et al., 2021; Wang et al., 2021). However, traditional luminogenic molecules commonly suffer from a troublesome luminescence “aggregation-caused quenching (ACQ)” phenomenon (Mei et al., 2015). Fortunately, in 2001, it was discovered by Tang and co-workers that 1-methyl-1,2,3,4,5-pentaphenylsilole showed an interesting “aggregation-induced emission (AIE)” effect, and emitted strong aggregative-state fluorescence (Luo et al., 2001). Subsequently, in 2002, Park et al. reported the “aggregation-induced emission enhancement (AIEE)” effect (An et al., 2002). Obviously, AIE or AIEE phenomenon is opposite to the pernicious ACQ, and AIE or AIEE-active luminophors are more conducive to practical applications. To date, a variety of luminogens possessing AIE or AIEE property have been reported (Dong et al., 2015; Chen et al., 2016; Yang et al., 2016; Ma et al., 2021; Yin et al., 2021). Among them, tetraphenylethene (TPE)-modified fluorescent molecules have received special attention from researchers due to their excellent aggregative-state emissive performance. When in the aggregates, the structurally twisted TPE molecules cannot pack in through a tight *π*-*π* stacking interactions network, and thus restrict the formation of ACQ effect. Meanwhile, the restricted intramolecular rotations block the non-radiative decays and open up the radiative channels.

Solvatochromism is an interesting phenomenon, which involves a color change process in connection with the solvent polarity. Studies of fluorescent dyes with solvatochromism are considered to be important, and solvatochromic luminogens can be used to evaluate solvent parameters. On the other hand, fluorescent organic molecules that are responsive to mechanical stimulus have attracted substantial interest because of their promising applications in mechanosensors and fluorescent switches (Chen et al., 2015; Chen et al., 2017; Chen et al., 2018; Chen et al., 2019; Wang et al., 2020; Zhang et al., 2020; Hu et al., 2021; Hu et al., 2021; Yin et al., 2021). In this work, we reported a TPE-modified rhodanine derivative (Chart 1), and the compound exhibited AIEE and mechanofluorochromic characteristics. Furthermore, this fluorescent molecule could selectively and sensitively detect Hg^2+^ in an acetonitrile solution and on test paper strips. Rhodanine-3-acetic acid is an electron acceptor, and the combination of the acceptor and TPE core provides a pull-push feature to the obtained target molecule. As a result, luminogen 1 also showed solvatochromic fluorescence behavior.

**CHART 1:** The molecular structure of TPE-modified rhodanine derivative 1.

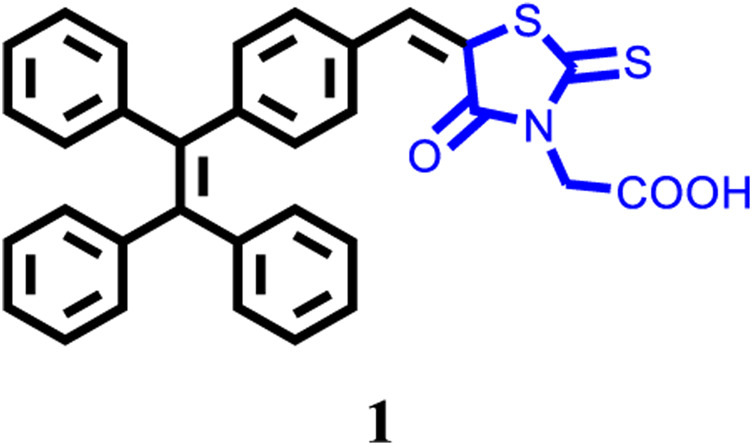

## Experimental Section

**SCHEME 1:**
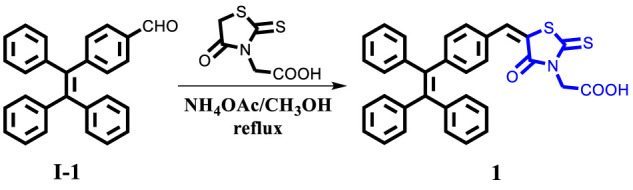
Synthesis of compound 1.

General methods: This target compound 1 was synthesized according to the route shown in **Scheme 1**. All synthetic experiments were performed under dry argon atmosphere applying standard Schlenk techniques. 2-(4-Oxo-2-thioxothiazolidin-3-yl)acetic acid and other starting materials and reagents were purchased from Innochem (Beijing, China) and used without further purification. Compound I-1 was synthesized according to the reported literature (Zhao et al., 2012). In addition, 1H NMR and 13C NMR spectra were collected on Bruker AVANCE NEO 500 MHz FT-NMR Spectrometer (500 MHz), chemical shift of internal standard tetramethylsilane at 0.00 ppm. Mass spectrum was collected by an ion trap MSD spectrometer (Agilent). Elemental analyses (C, H, N) were carried out with a PE CHN 2400 analyzer. Ultraviolet/Visible absorption spectra were measured on an Agilent 8453 UV-Vis spectrophotometer. Emission spectra were recorded on a Hitachi-F-4600 ﬂuorescence spectrophotometer or an Edinburgh FLS1000 ﬂuorescence spectrometer. DLS datum was recorded by NanoBrook 90Plus (Brookhaven Instruments). Fluorescence images were collected on an Olympus FV1000 confocal laser scanning microscope. Powder XRD experiments were carried out by Shimadzu XRD-6000 diffractometer with Ni-ﬁltered and graphite-monochromated Cu K_a_ radiation (*λ* = 1.54 Å, 40 kV, 30 mA).

Synthesis of compound 1: A 100 ml two-necked round-bottom flask was evacuated under vacuum and then flushed with dry argon three times. Subsequently, compound I-1 (720.92 mg, 2.0 mmol), 2-(4-Oxo-2-thioxothiazolidin-3-yl)acetic acid (2.0 mmol), NH_4_OAc (2.0 mmol), and methanol (60 ml) were added into the round-bottom flask. After that, the mixture was reﬂuxed for 24 h and then cooled to room temperature. After the disappearance of the starting materials, monitored by TLC board, the resulting mixture was extracted with dichloromethane and water, and then washed with brine three times. Organic layer was dried by anhydrous MgSO_4_, then the organic solvent was removed under vacuum, the residues were purified by column chromatography on silica gel, affording the corresponding target product 1 (yellow solid) in a yield of 62.6%. 1: 1H NMR (500 MHz, DMSO-d6): *δ* (ppm) = 13.48 (s, 1H), 7.79 (s, 1H), 7.46 (d, *J* = 5 Hz, 2H), 7.18–7.13 (m, 11H), 7.02–6.97 (m, 6H), 4.73 (s, 2H). 13C NMR (125 MHz, DMSO-*d*6): *δ* (ppm) = 193.2, 167.4, 166.5, 146.5, 142.9, 142.8, 142.7, 142.3, 139.7, 133.5, 131.9, 131.0, 130.9, 130.8, 130.7, 130.6, 128.2, 128.0, 127.1, 127.0, 121.5, 45.1. HRMS (m/z): 533.1113 [M]^+^ (calcd 533.1119). Anal. Calcd. For C_32_H_23_NO_3_S_2_: C, 72.02; H, 4.34; N, 2.62. Found: C, 72.15; H, 4.25; N, 2.67.

## Results and Discussion

**FIGURE 1:**
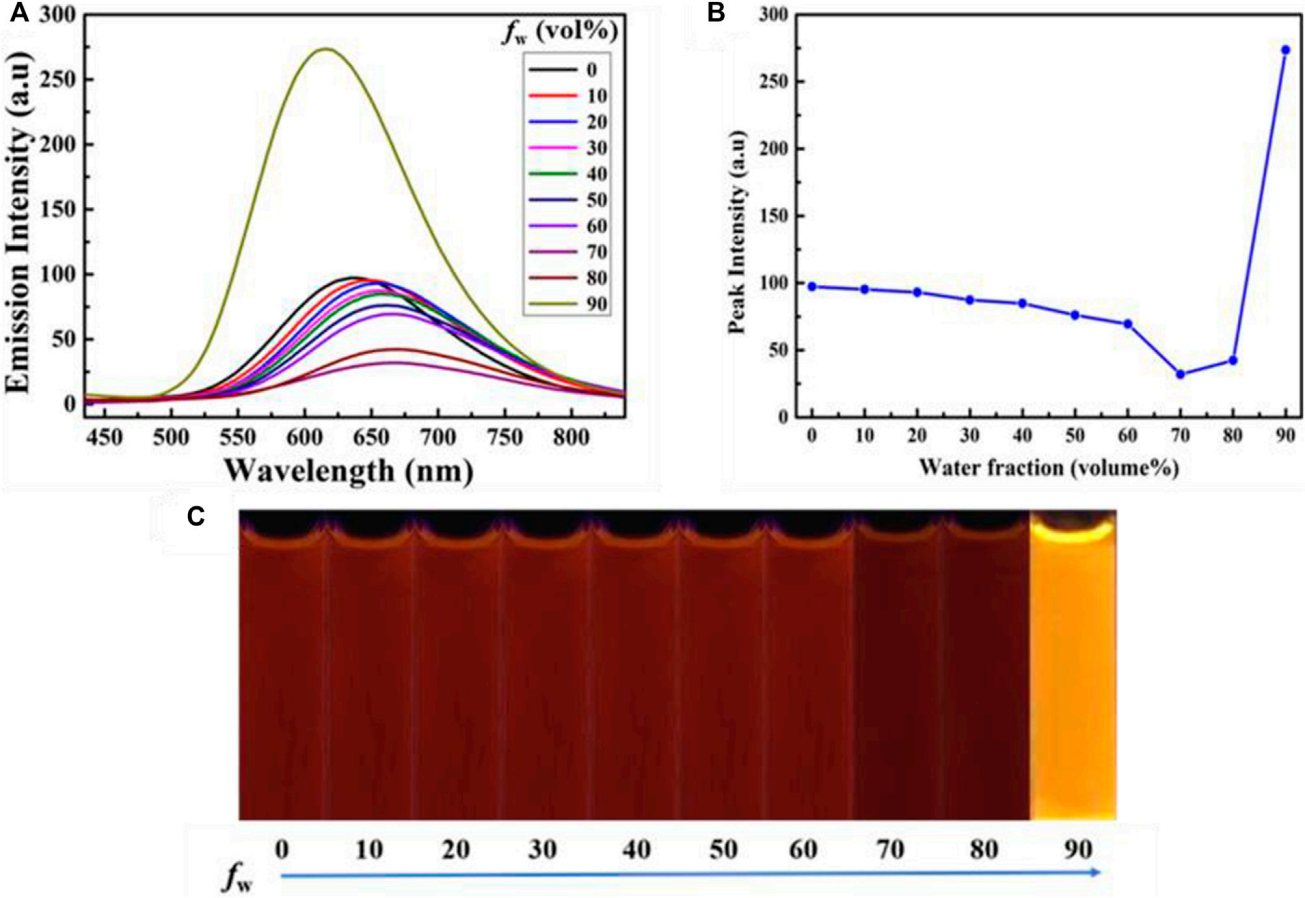
**(A)** Emissive spectra of the dilute solutions of compound 1 (concentration: 2.0 × 10^−5^ mol L^−1^) in CH_3_CN-H_2_O mixtures with varying *f*_w_ values. **(B)** Changes in the emission intensity of 1 at 618 nm in CH_3_CN-H_2_O mixtures with varying *f*_w_ values (0–90%). **(C)** PL images of 1 (20 μM) in CH_3_CN-H_2_O mixtures with varying *f*_w_ values (0–90%) under 365 nm UV light.

In order to investigate the aggregative-state emissive behavior of compound 1, the UV-Vis absorption spectra of compound 1 (20 μM) in CH_3_CN-H_2_O mixtures with different water fractions were initially measured. Obviously, level-off tails were noticed in the long-wavelength region as the water content increased (Supplementary Figure S1). Such a phenomenon indicated the formation of nano-aggregates (Leung et al., 2013). Next, the photoluminescence (PL) spectra of luminogenic molecule 1 (20 μM) in CH_3_CN-H_2_O mixtures with various volume fractions of water (*f*_w_) were studied. As presented in Figure 1, luminogen 1 (20 μM) in pure CH_3_CN displayed weak red ﬂuorescence due to an intramolecular charge transfer (ICT) from the TPE unit to the rhodanine-3-acetic acid unit, the fuorescence quantum yield of luminogen 1 (20 μM) in pure CH3CN was 0.7%, and the emission intensity of red ﬂuorescence gradually weakened when the *f*_w_ value was continuously increased to 70%, which was attributed to the twisted ICT effect (Shen et al., 2012; Liu et al., 2019). When the *f*_w_ value was 90%, the quantum yield of luminogen 1 in the CH_3_CN-H_2_O mixture was 6.6%.

**FIGURE 2:**
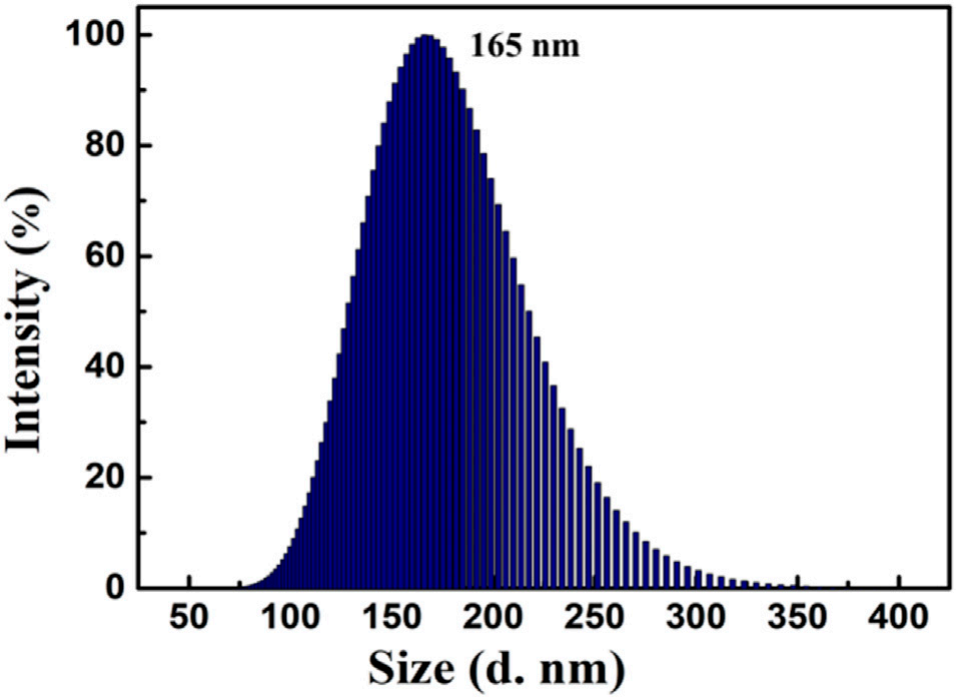
Size distribution curve of compound 1 (2.0 × 10^−5^ mol L^−1^) in CH_3_CN-H_2_O mixture with 90% water fraction.

Interestingly, when the water content reached 90%, a bright orange ﬂuorescence was observed. Water is a non-solvent for luminogen 1, increasing the *f*_w_ value in the mixed solvent leading to the transition of the compound from a well-dispersed state in pure CH_3_CN to aggregated particles in CH_3_CN-H_2_O mixture with 90% water fraction. Indeed, the nano-aggregates obtained were confirmed by carrying out dynamic light scattering (DLS) experiment (Figure 2). The strong orange ﬂuorescence of luminogen 1 was thus triggered by aggregation, and compound 1 exhibited typical AIEE feature.

**FIGURE 3:**
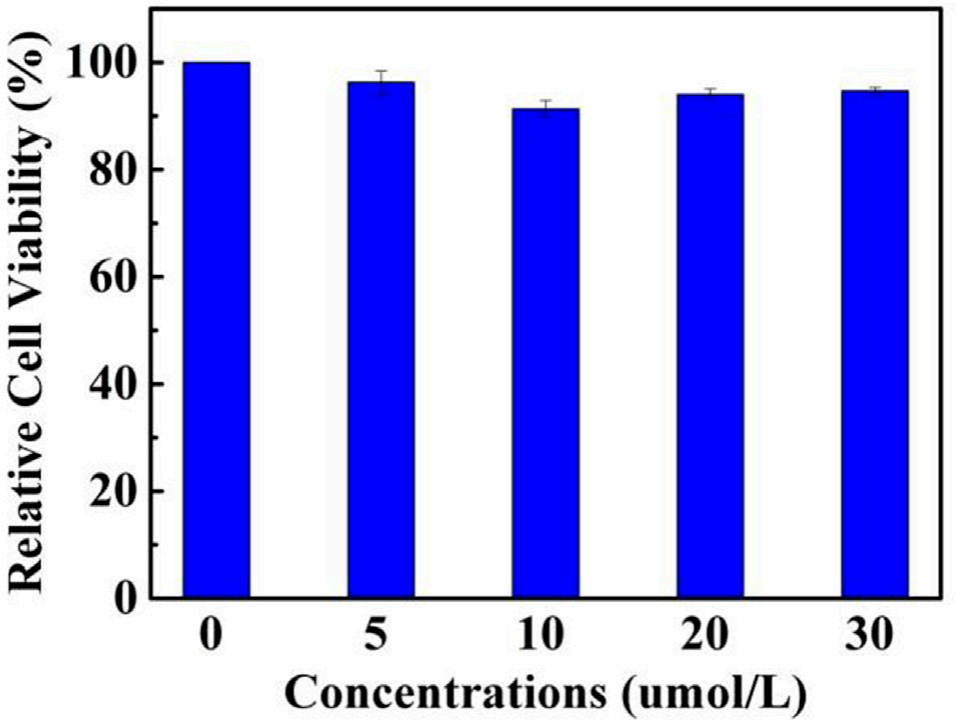
The MTT assay of rhodanine derivative 1 for measuring cell viability.

**FIGURE 4:**
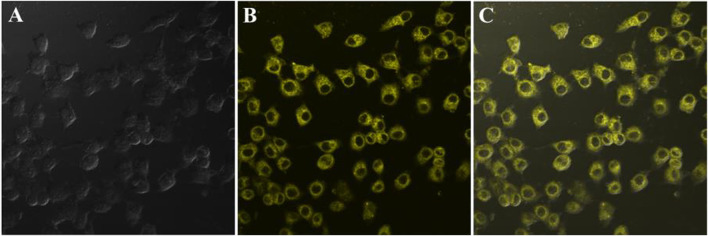
Fluorescence images of HeLa cells incubated with rhodanine derivative 1 (20 μM) for 30 min at 37°C; from left to right are bright ﬁeld image **(A)**, fluorescence image **(B),** and merge image **(C)**.

In consideration of the remarkable AIEE nature of compound 1, the prepared fluorescent molecule was applied for cell imaging. HeLa cells were chosen as testing cells to culture and stain with luminogen 1, and the cell imaging effect was evaluated by a confocal laser scanning microscopy (CLSM). The standard MTT experiment suggested that the TPE-modified rhodanine derivative displayed low cytotoxicity (Figure 3). Subsequently, HeLa cells were incubated with AIEE-active compound 1 (20 μM) for 30 min at 37°C and the fluorescence images were collected by CLSM. As demonstrated in Figure 4, an intense yellow fluorescence was observed inside the cells. In addition, picture C, which is the overlap of pictures A and B, indicated that rhodanine derivative 1 possessed good cell imaging effect.

**FIGURE 5:**
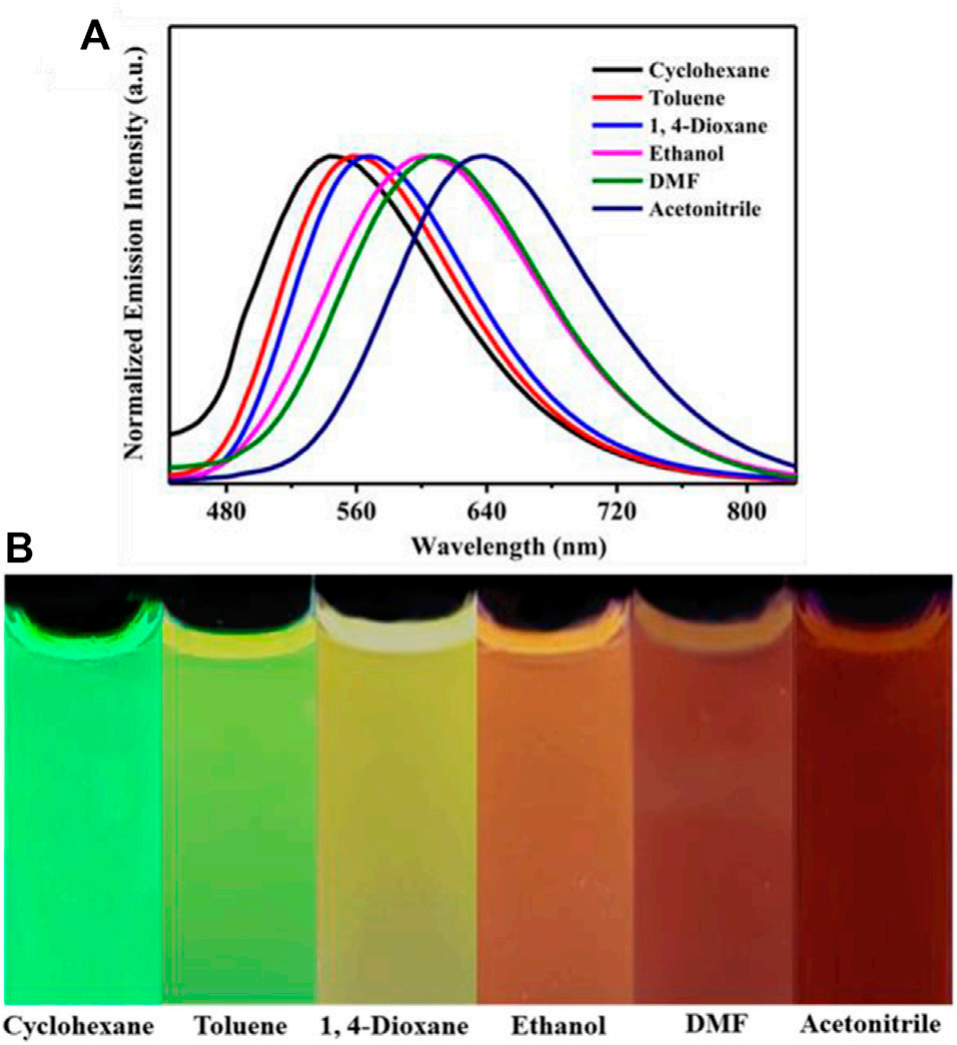
**(A)** Normalized fluorescence spectra (Excitation wavelength = 365 nm) of compound 1 (2.0 × 10^−5^ mol L^−1^) in different solvents. **(B)** PL images of compound 1 (2.0 × 10^−5^ mol L^−1^) in different solvents under 365 nm UV illumination.

**FIGURE 6:**
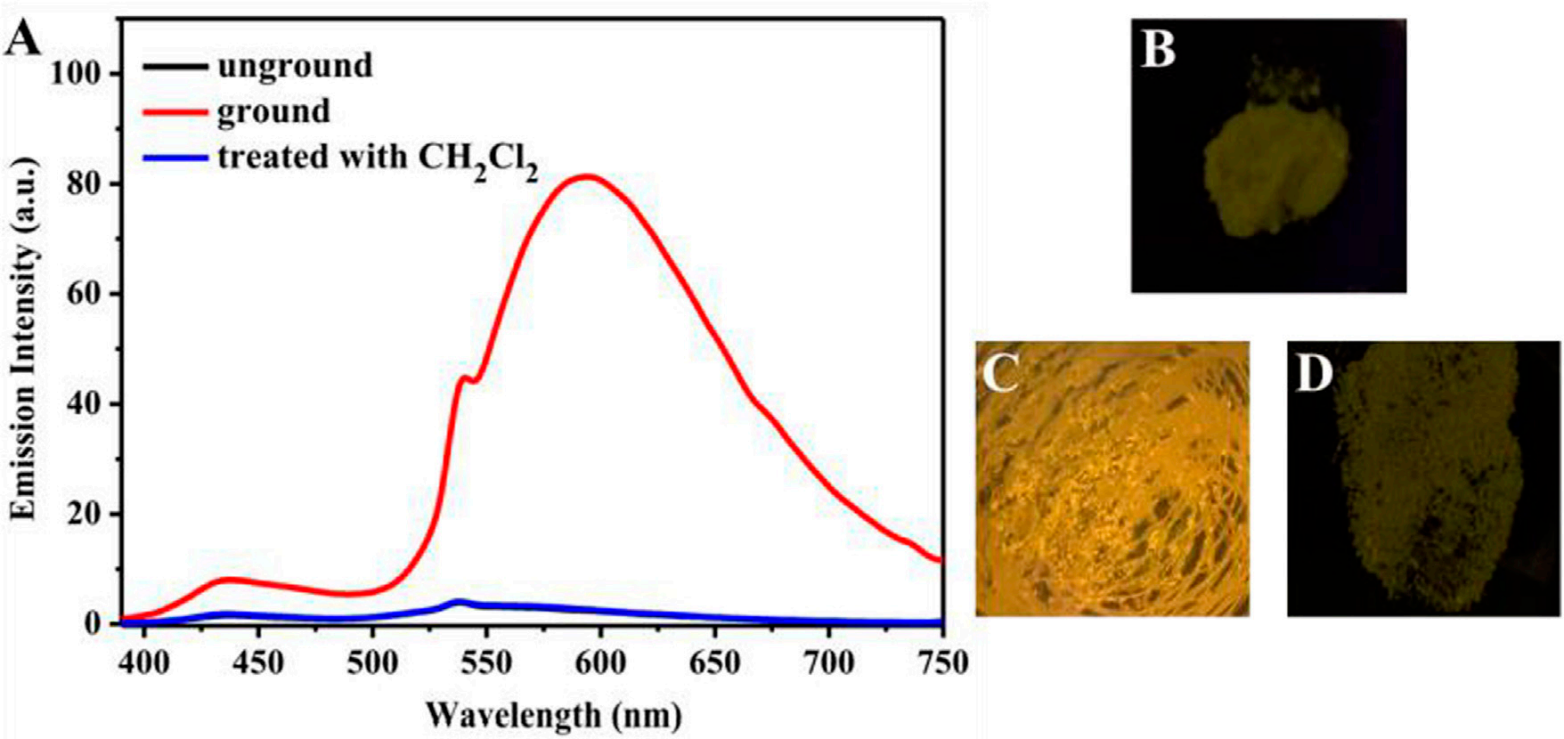
**(A)** Fluorescence spectra of solid sample 1 before grinding, after grinding, after treatment with dichloromethane vapor. The excitation wavelength is 365 nm. Fluorescence images of sample 1 in various states under 365 nm UV light: **(B)** as-synthesized sample, **(C)** ground sample, **(D)** sample after treatment with dichloromethane vapor.

Introduction of electron acceptor rhodanine-3-acetic acid group to a TPE group may endue the target compound with not only the ICT effect but also with the solvatochromic behavior. As shown in Figure 5, luminogen 1 (20 μM) displayed various fluorescent colors, including green, yellow-green, yellow, orange, and red, in a series of solvents with increasing polarities (Cyclohexane < Toluene <1,4-Dioxane < Ethanol < DMF < Acetonitrile). A striking red shift of the emission peak from 535 to 640 nm was recorded with the increasing solvent polarities. And polarity may be the dominant reason for the large red shift (Shen et al., 2012). We mainly studied the solvent polarity and the red shift of the emission peak, so we completed a uniform treatment of the intensity of the largest emission peak in different solvents, which was convenient and straightforward for this study. In a word, these results indicated that the fluorescence behavior of luminogen 1 was sensitive to solvent polarity, exhibiting strong solvatochromism phenomenon. In addition, as can be seen in Figure 6, the pristine solid sample 1 emitted weak yellow fluorescence. However, upon grinding, an obvious orange emission band with a *λ*_max_ at 594 nm was observed, and a bright orange ﬂuorescence could be seen. When the orange-emitting sample was fumed with dichloromethane vapor for 30 s, the ﬂuorescent color rapidly changed back to faint yellow. Therefore, luminogen 1 also exhibited reversible mechanochromic fluorescence feature.

**FIGURE 7:**
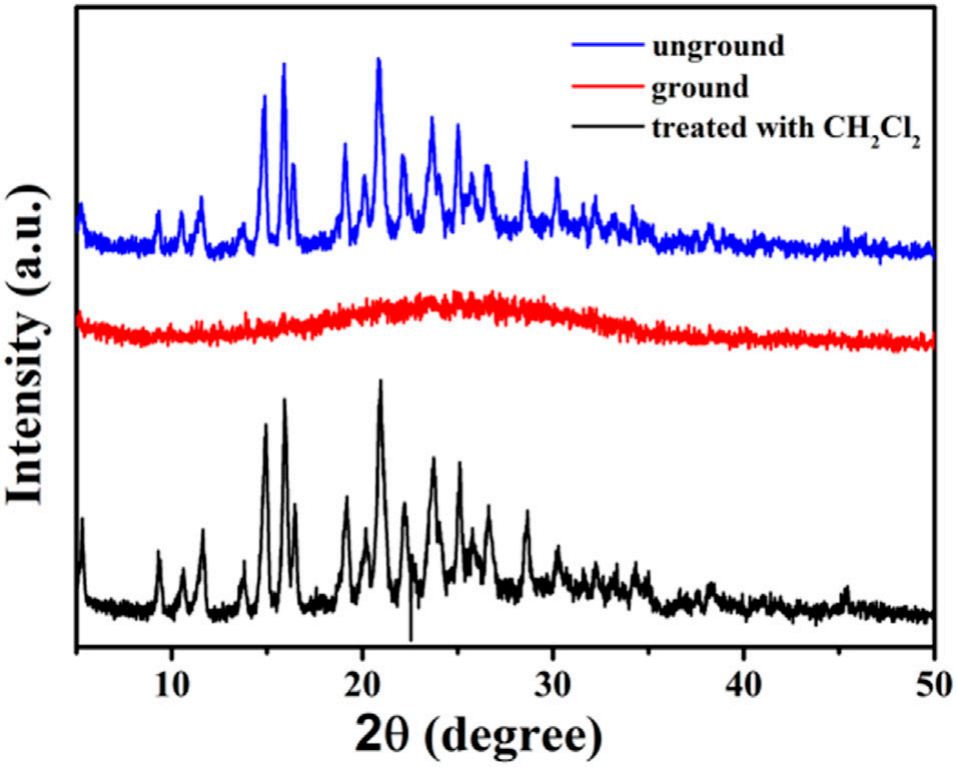
XRD patterns of compound 1 in different solid states: unground, ground, and after treatment with dichloromethane solvent vapor.

By analyzing the obtained powder X-ray diffraction (PXRD) patterns of compound 1 before and after grinding (Figure 7), it was discovered that the fluorescent change from weak yellow emission to strong orange emission was attributed to crystalline-to-amorphous morphological transformation.

The colorimetric sensing ability of compound 1 toward Hg^2+^ was evaluated using UV-Vis absorption spectroscopy. Changes in the UV-Vis absorption spectra of 1 (20 μM) in acetonitrile upon addition of Hg^2+^ were shown in Supplementary Figure S2. After the concentration of Hg^2+^ was increased to 6 eq., a new absorption band with a maximum at 365 nm was observed. At the same time, the visible color of the solution changed from pale yellow to colourless. The ﬂuorescence response of 1 to Hg^2+^ was illustrated in Supplementary Figure S3. Obviously, the fluorescence intensity of 1 gradually decreased as the Hg^2+^ concentration progressively increased from 0 to 10.0 equivalents, and the emissive color was transformed from red to colorless, and then followed by a plateau via further titration (Supplementary Figure S4). Based on the titration experiments, we calculated the detection limit of TPE-modified rhodanine derivative 1 for Hg^2+^, which was 6.0 × 10^−7^ mol L^−1^ (Supplementary Figure S5). This quenching constant of 1 with Hg^2+^ was calculated to be 9.74 × 10^4^ M^−1^ (Supplementary Figure S6). Furthermore, the Job’s plot also demonstrated 1:1 stoichiometric ratio between 1 and Hg^2+^ (Supplementary Figure S7). Moreover, the binding interaction of compound 1 and Hg^2+^ was surveyed by 1H NMR in dimethyl sulfoxide-*d*_6_. Apparently, 1 exhibited a singlet at 13.48 ppm of the carboxyl hydrogen (COOH) (Supplementary Figure S8), and the singlet at 13.48 ppm disappeared with new peaks at 4.3 and 7.8 ppm appeared when the analyte concentration was 10 equiv. We speculated that the carbonyl group on the rhodanine structure and the hydroxyl group of the carboxylic acid were combined with Hg^2+^, which destroyed the original *p* conjugation and most of the *π* electron density would be transferred to the carbonyl carbon, thus causing a corresponding shift in the mass spectrum (Thamaraiselvi et al., 2019). Furthermore, the binding interaction of compound 1 and Hg^2+^ (Hg^2+^ solution prepared with DMSOd) was surveyed by ^1^H NMR in dimethyl sulfoxide-*d*_6_ again (Supplementary Figure S8B). On the basis of Pearson’s Hard-Soft Acid-Base Theory (Ralph 1993), Hg^2+^ can interact preferentially with sulfur, oxygen, and nitrogen. Besides, to further assess the binding mode of 1 toward Hg^2+^, the probe was used to detect its molecular mass with 10.0 equiv. Hg^2+^, the exact value at m/z = 833.2428 showed high affinity for Hg^2+^ combine [1 + H^+^+ Hg^2+^ + NO_3_^−^ + Cl^−^]^+^, which indicated the binding of 1 with Hg^2+^ with 1 : 1 stoichiometry (Supplementary Figure S9).

**FIGURE 8:**
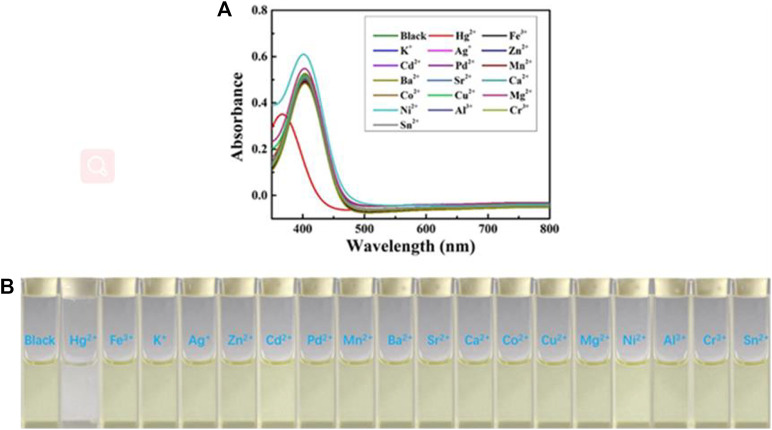
**(A)** The UV-Visible spectra of chemosensor 1 (concentration: 2.0 × 10^−5^ mol L^−1^) in acetonitrile upon addition of 10 equiv. various metal ions. **(B)** Color changes of chemosensor 1 (concentration: 2.0 × 10^−5^ mol L^−1^) in acetonitrile upon addition of 10 equiv. various metal ions.

**FIGURE 9:**
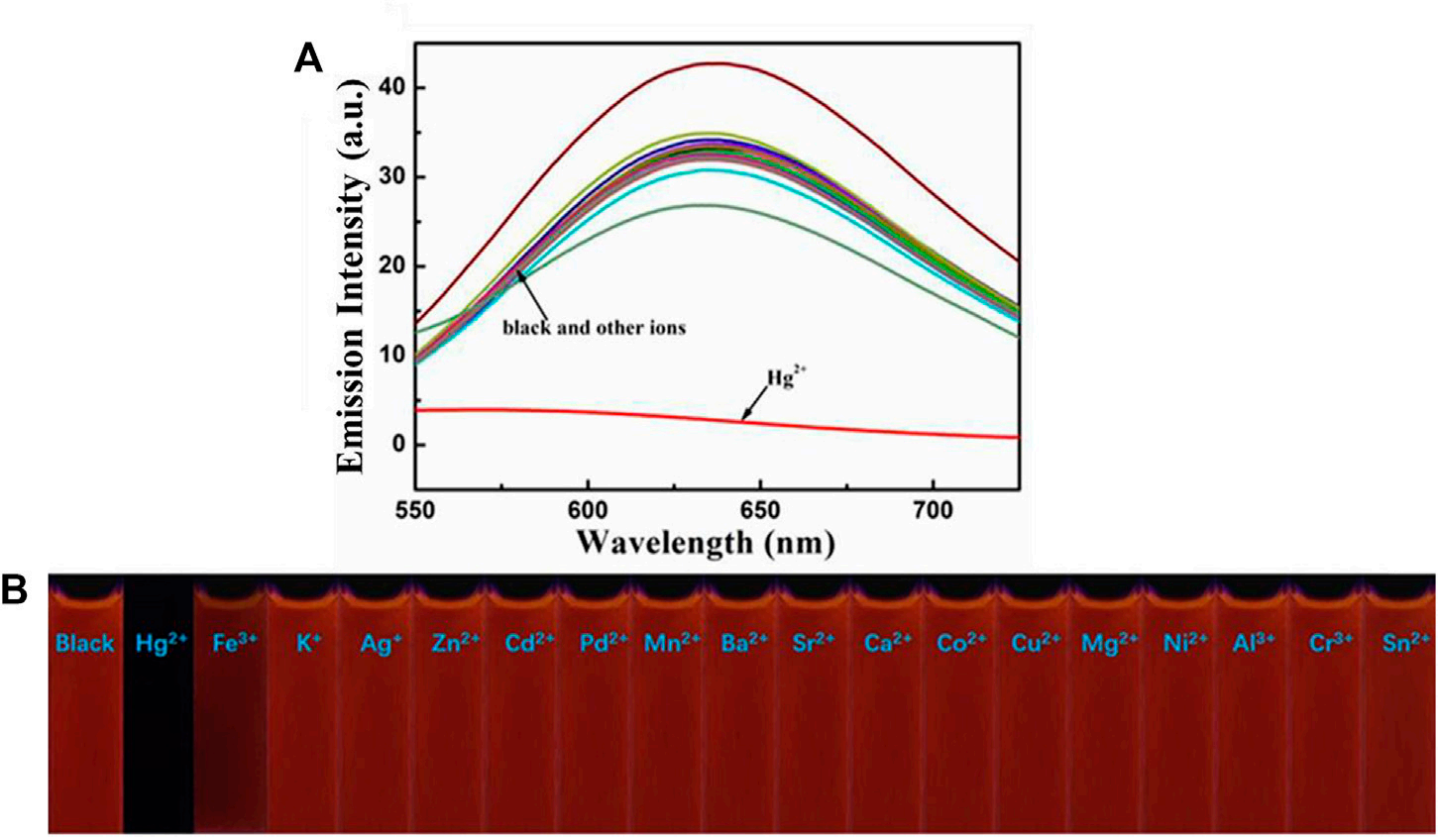
**(A)** Fluorescence spectra of compound 1 (2.0 × 10^−5^ mol L^−1^) in acetonitrile towards various cations. Excitation wavelength = 425 nm. **(B)** Fluorescence photographs of compound 1 (2.0 × 10^−5^ mol L^−1^) in acetonitrile after addition of various metal ions (10.0 equiv.) under 365 nm UV light.

Next, the sensing capabilities of compound 1 were examined in acetonitrile through the addition of various cations, including Hg^2+^, Fe^3+^, K^+^, Ag^+^, Zn^2+^, Cd^2+^, Pd^2+^, Mn^2+^, Ba^2+^, Sr^2+^, Ca^2+^, Co^2+^, Cu^2+^, Mg^2+^, Ni^2+^, Al^3+^, Cr^3+^, and Sn^2+^. As demonstrated by Figure 8, when 10.0 equivalents of metal ions listed above were added to the solution, only Hg^2+^ caused a color change from pale yellow to colourless. The UV-Vis absorption spectrum of the diluted acetonitrile solution containing compound 1 with mercury ion showed a clear blue-shifted absorption peak at 365 nm, and the absorption peaks of the solution with other metal ions and blank probes were all at 410 nm. It can be shown that the selective recognition of compound 1 and mercury ion can be realized in the UV-Vis absorption spectra. Presumably, it was attributed to the structural changes caused by the selective complexation of fluorescent probe 1 with mercury ion. Meanwhile, as showed in Figure 9, the red fluorescence was quenched by the addition of Hg^2+^ (10.0 equiv.). Before and after addition of mercury ion, the fluorescence quantum efficiency of compound 1 (2.0 × 10^−5^ mol L^−1^) in acetonitrile were 0.7 and 0.2%, respectively. The fluorescence spectrum of the dilute solution of acetonitrile containing compound 1 showed that the intensity of the emission peak at 625 nm was significantly reduced after adding 10 equivalents of mercury ion. However, when other metal cations were added separately into the solution containing compound 1, no notable fluorescent changes could be seen. It was inferred that the fluorescence probe 1 was complexed with mercury ion, which caused the structure of the fluorescence probe 1 to change, thus showing the effect of fluorescence “on-off.” This showed that the fluorescent probe 1 had excellent specific selectivity to mercury ion. Furthermore, as presented in Supplementary Figure S10, no remarkable interference was noticed when Hg^2+^ (10.0 equiv.) was added with other ions (10.0 equiv.).

**FIGURE 10:**
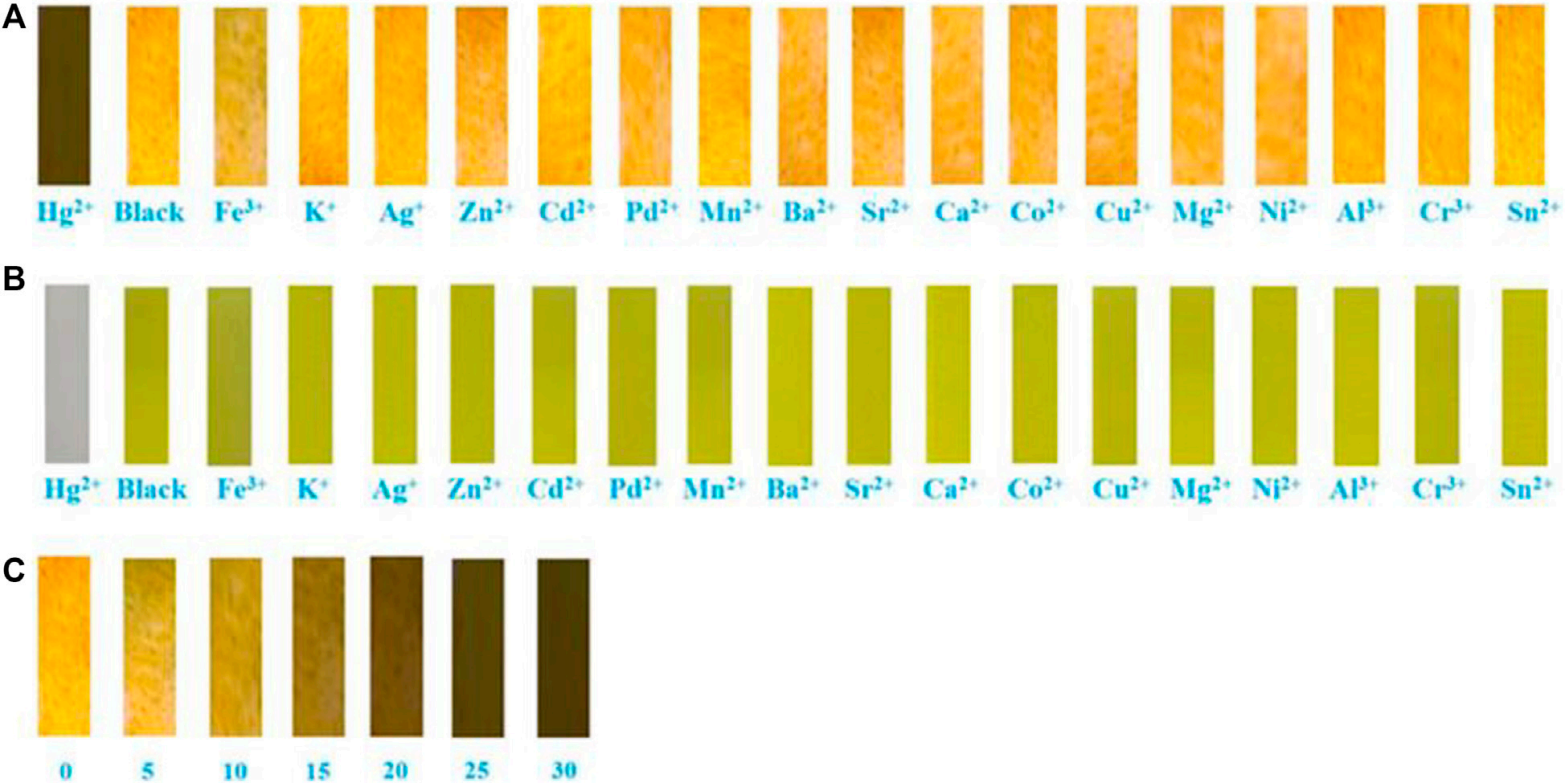
Photographs showing: **(A)** Fuorescent color changes of compound 1 on test paper strips after treating with various metal ions under 365 nm UV light. **(B)** Visible color changes of compound 1 on test paper strips after treating with various metal ions under natural light. **(C)** Fuorescent color changes of different concentrations of Hg^2+^ (2.0 × 10^−5^ mol L^−1^) on test paper strips.

Finally, the test strips were made for the detection of Hg^2+^. Specifically, we added test strips with the same size to the acetonitrile solutions of chemosensor 1 containing different metal ions. After 15 s, they were taken out from the solutions and then dried in air naturally. Obviously, Figure 10B shows that the test strip containing 1 and Hg^2+^ hardly emitted fluorescence under 365 nm UV light, while the test strips containing 1 and other metal ions exhibited orange fluorescence, which was consistent with that of the blank test strip. Similarly, the test strip containing 1 and Hg^2+^ was almost colorless under natural light (Figure 10B), and the blank test strip and test strips containing 1 and other metal ions were yellow under natural light. Furthermore, as can be seen in Figure 10C, the ﬂuorescent color of the test strip gradually changed from orange to colourless with the concentration of Hg^2+^ raising from 0 to 30 × 10^−5^ mol L^−1^. In order to study the effect of pH on compound 1, we tested the fluorescence characteristics of compound 1 in the range of pH (2–13). As depicted in Supplementary Figure S13B, the fluorescence intensity of compound 1 at 649 nm was relatively stable in the range of pH 3–7. With the increase of pH, the fluorescence intensity of the blank probe gradually decreased, which was caused by the influence of the carboxylic acid on the rhodanine structure under alkaline conditions. The experimental results showed that compound 1 could be tested under acidic and neutral conditions.

## Conclusion

In summary, a TPE-modified rhodanine derivative 1 was reported, and the compound with ICT effect exhibited typical AIEE and good cell imaging properties. Interestingly, the luminogenic molecule not only displayed solvatochromic fluorescence behavior but also displayed reversible mechanochromic fluorescence phenomenon. In addition, luminogen 1 could selectively and sensitively detect Hg^2+^ in an acetonitrile solution. Meanwhile, luminogen 1 could also be applied to detect Hg^2+^ on test paper strips. This work provides a valuable reference to the preparation of TPE-based multifunctional luminophors.

## Data Availability

The original contributions presented in the study are included in the article/Supplementary Material, further inquiries can be directed to the corresponding authors.
