# Supplementary material for: Tetraphenylethene-Modified Colorimetric and Fluorescent Chemosensor for Hg2+ With Aggregation-Induced Emission Enhancement, Solvatochromic, and Mechanochromic Fluorescence Features
Source: Front Chem. 2022 Jan 26;9:811294. doi: 10.3389/fchem.2021.811294 (PMC8828043; doi:10.3389/fchem.2021.811294)
Supplement: Supplementary file 1 [file DataSheet1.doc]

**Supplementary Information**

**Tetraphenylethene (TPE)-modified colorimetric and fluorescent chemosensor for Hg^2+^ with aggregation-induced emission enhancement (AIEE), solvatochromic and mechanochromic fluorescence features**

*Jin-jin Tian ^‡,1^, Dian-dian Deng ^‡,1^, Long Wang ^1^, Zhao Chen ^1*^ and Shouzhi Pu ^1,2*^*

*^1^ Jiangxi Key Laboratory of Organic* *Chemistry, Jiangxi Science and Technology Normal University, Nanchang 330013, PR China*

*^2^* *Department of Ecology and Environment, Yuzhang Normal University, Nanchang 330103, PR China*

*E-mail addresses:* [*chenzhao666@126.com*](mailto:chenzhao666@126.com) *(Z. Chen),* [*pushouzhi@tsinghua.org.cn*](mailto:pushouzhi@tsinghua.org.cn) *(S. Pu).*

*^‡^ These authors contributed equally to this work.*

**Table of Contents**

1. **FIGURES. S1-S13………………………………............................................S3**
2. **Copies of NMR spectra and Mass spectrum..................................................S10**
3. **FIGURES. S1-S13**


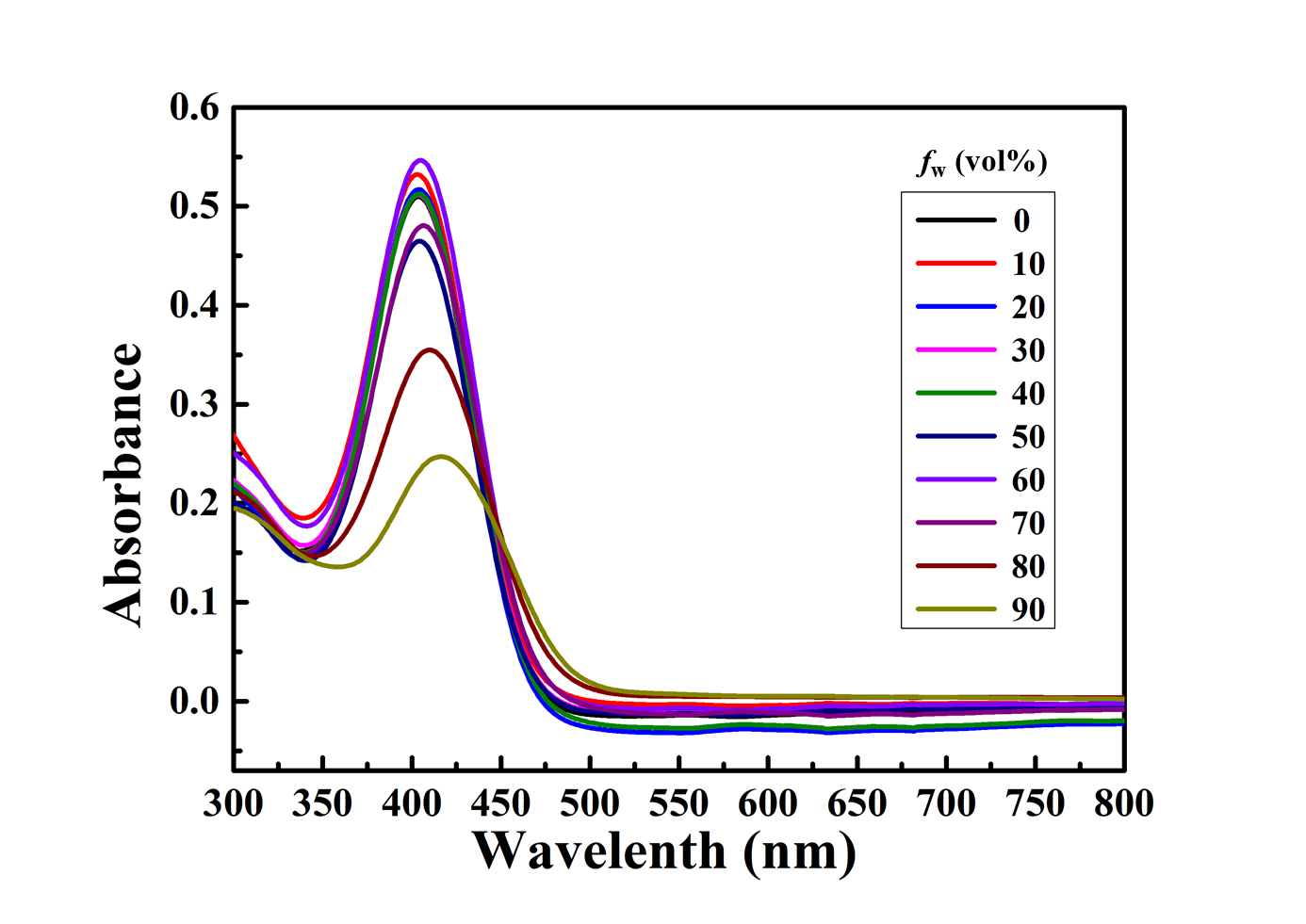


**FIGURE S1 |** UV-Vis absorption spectra of compound **1** (concentration: 2.0 × 10^-5^ mol L^-1^) in CH_3_CN-H_2_O mixtures with varying *f*_w_ values.


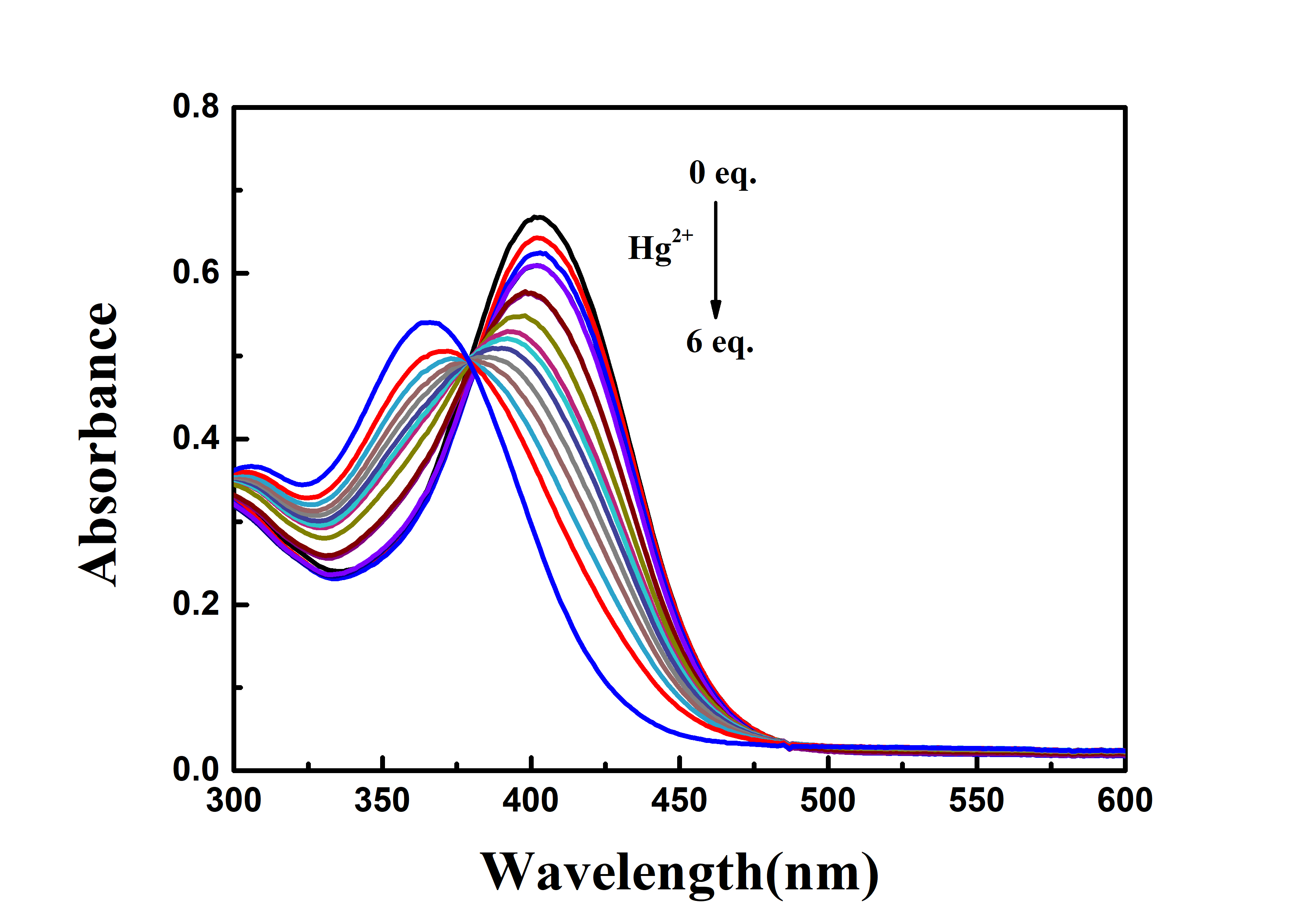


**FIGURE S2 |** Absorbance titration spectra of compound **1** (concentration: 2.0 × 10^-5^ mol L^-1^) induced by Hg^2+^ (0-6.0 equiv.) in an acetonitrile solution.


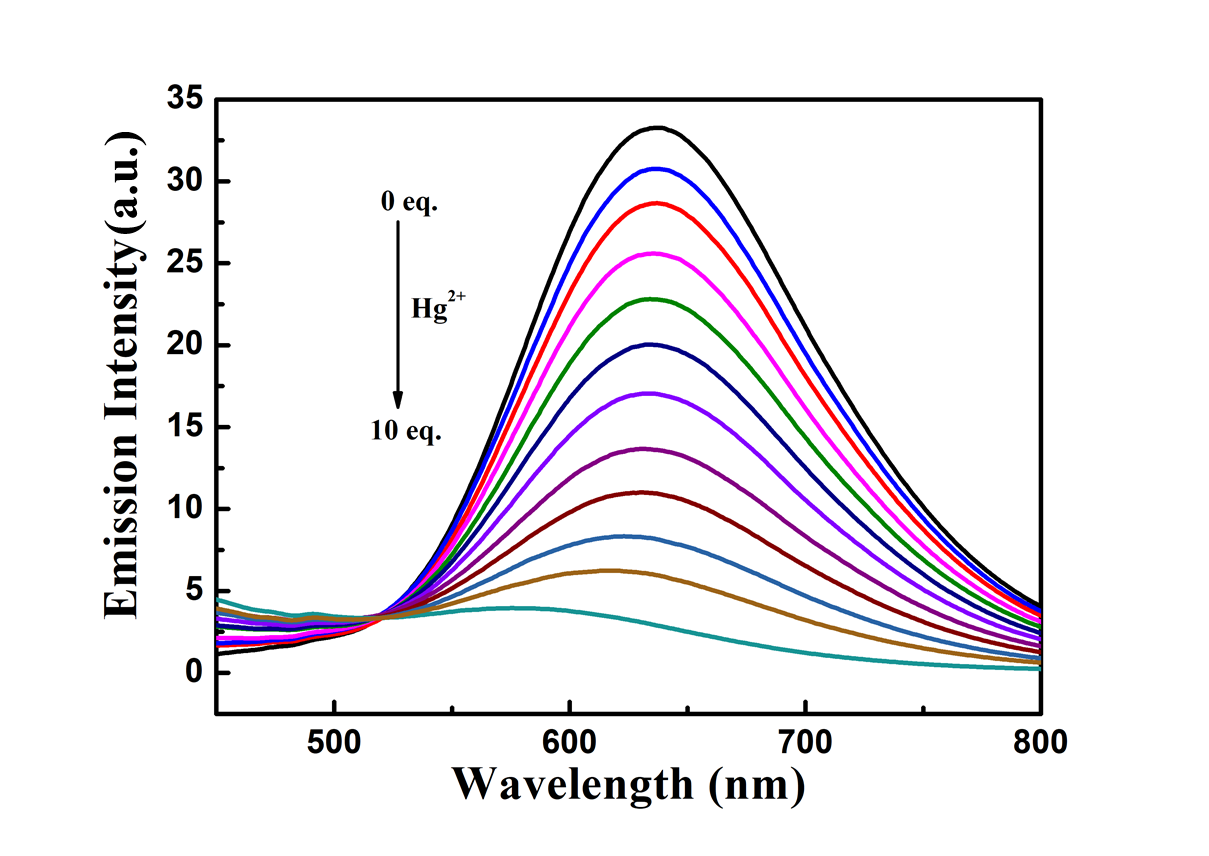


**FIGURE S3 |** Fluorescence titration spectra of compound **1** (concentration: 2.0 × 10^-5^ mol L^-1^) induced by Hg^2+^ (0-10.0 equiv.) in an acetonitrile solution. Excitation wavelength = 425 nm Excitation slit = 5 Emission slit = 5.


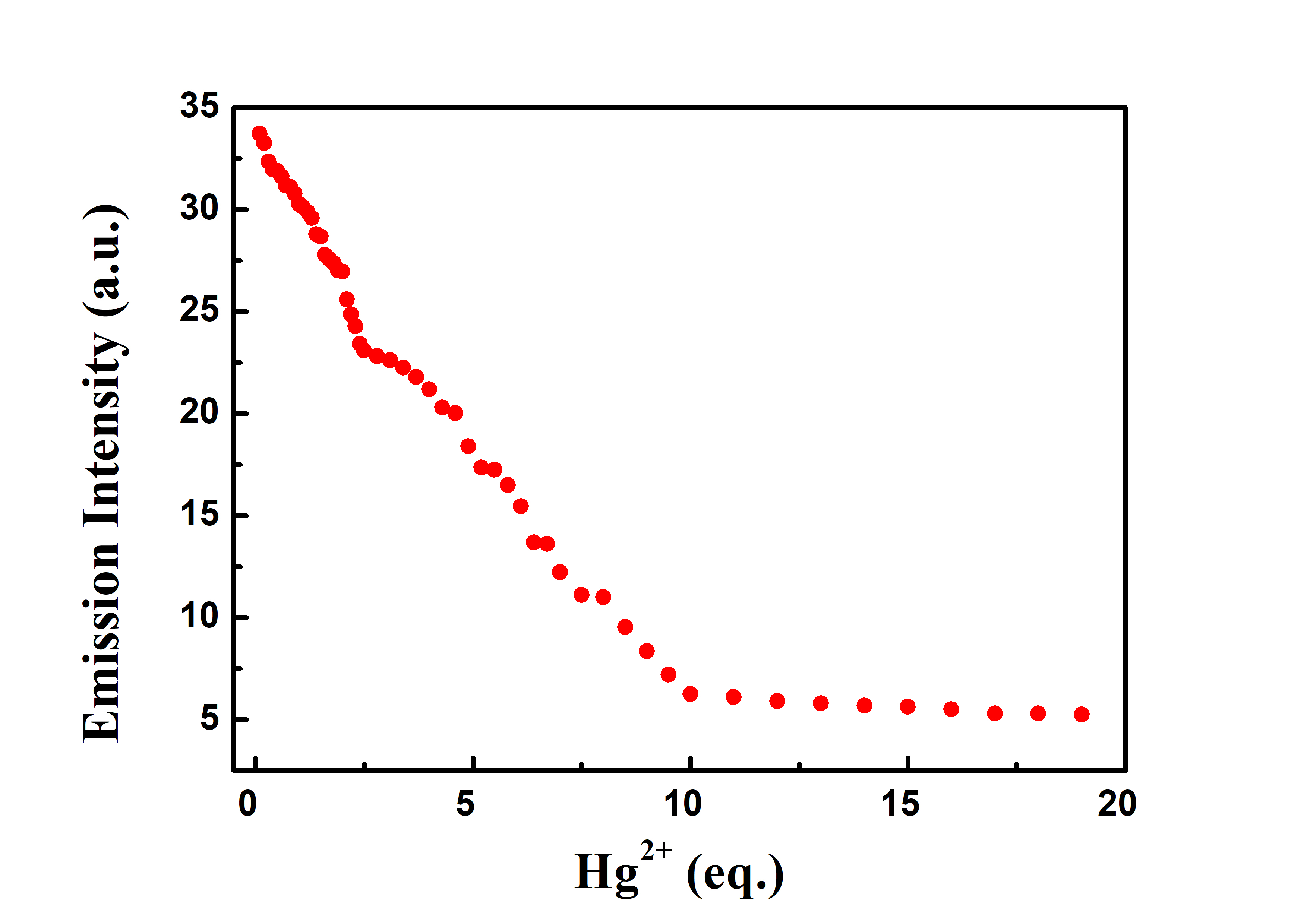


**FIGURE S4 |** The emission intensity changes of compound **1** at 625 nm with different equivalents of Hg^2+^.


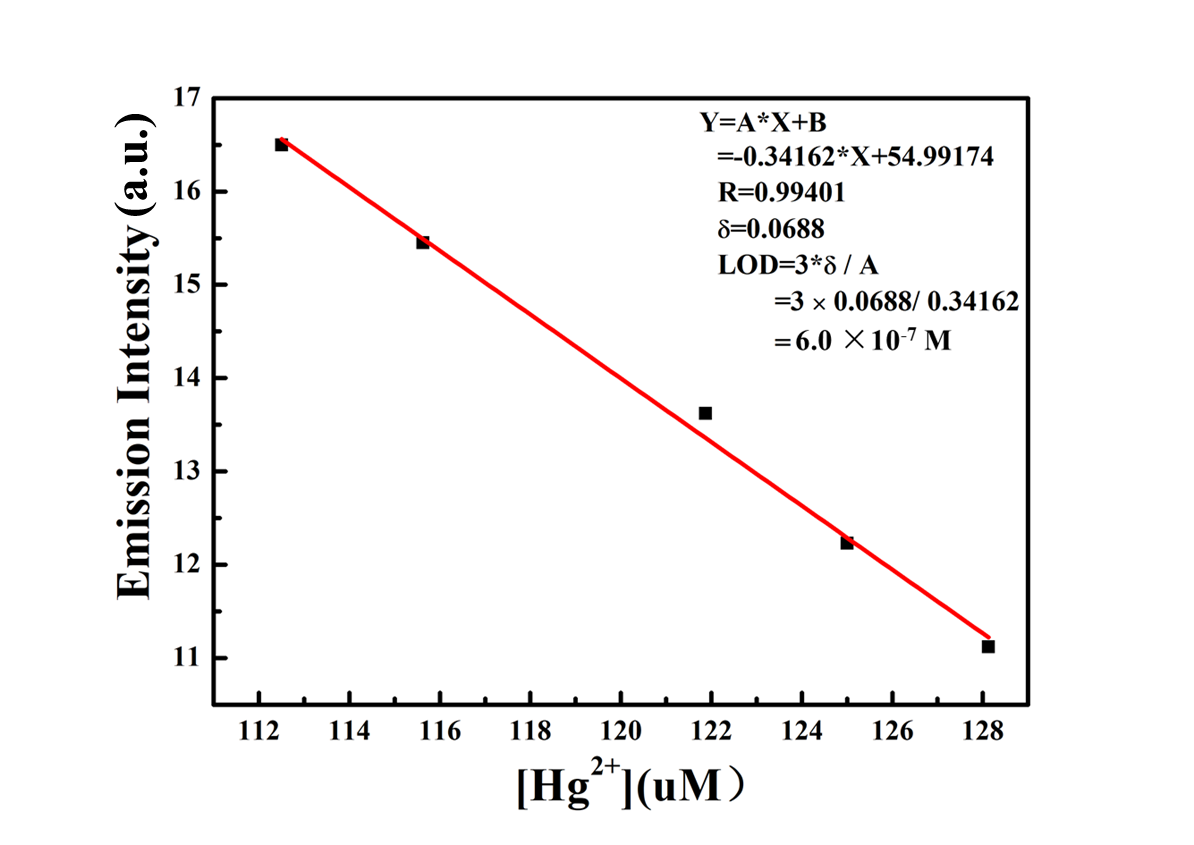


**FIGURE S5 |** The limit of detection (LOD), LOD is 6.0 × 10^-7^ M.


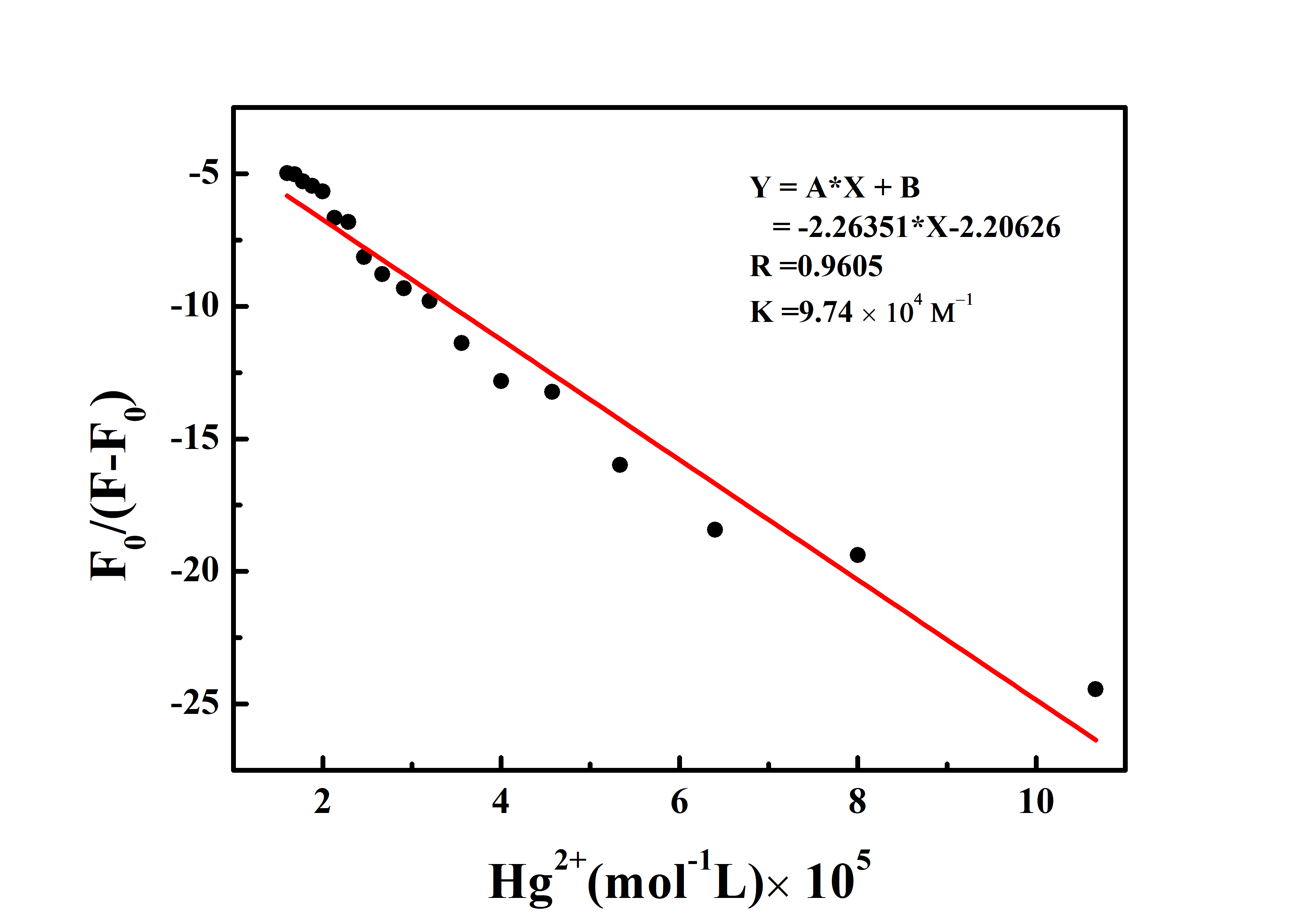


**FIGURE S6 |** Hildebrand-Benesi plot based on the 1 : 1 ratio for compound **1** and Hg^2+^ , the binding constant is 9.74 × 10^4^ M^–1^.


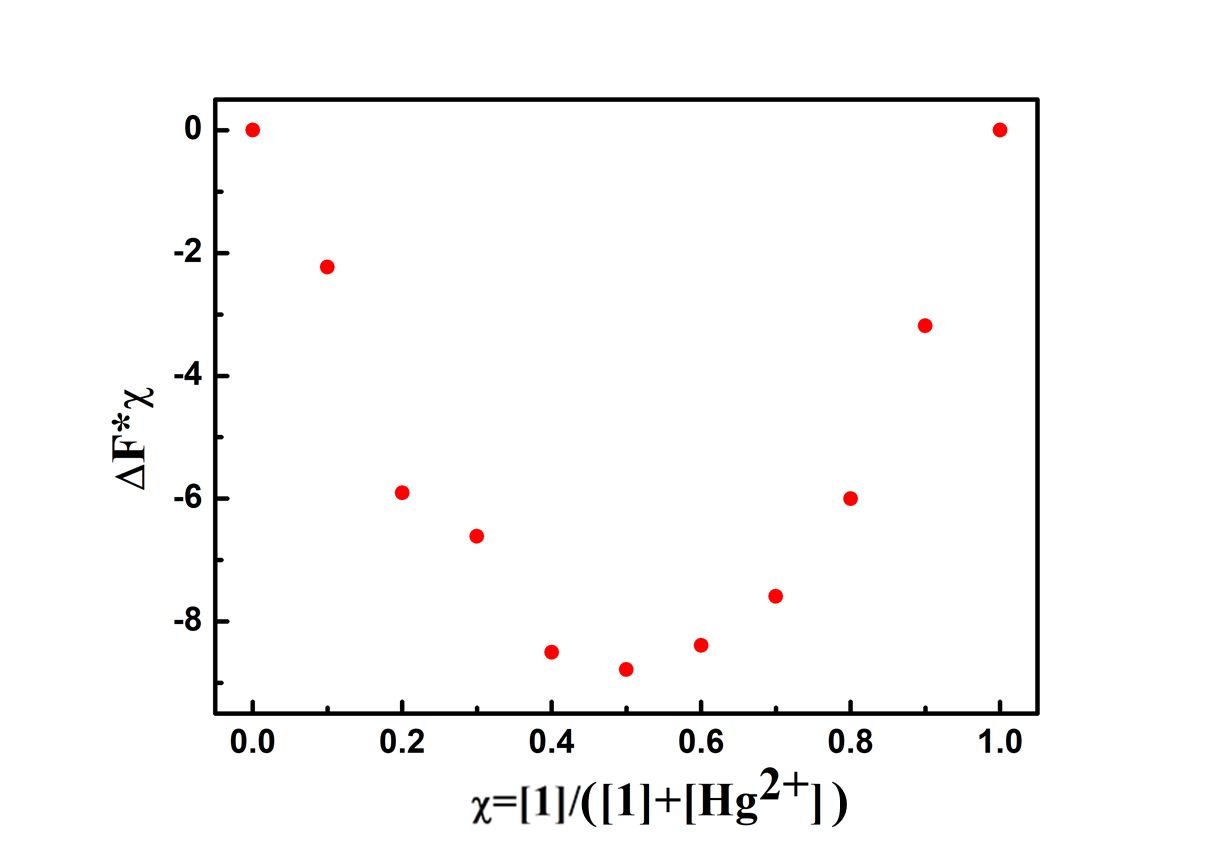


**FIGURE S7 |** Job’s Plot of compound **1** with Hg^2+^ showing 1: 1 stoichiometry.


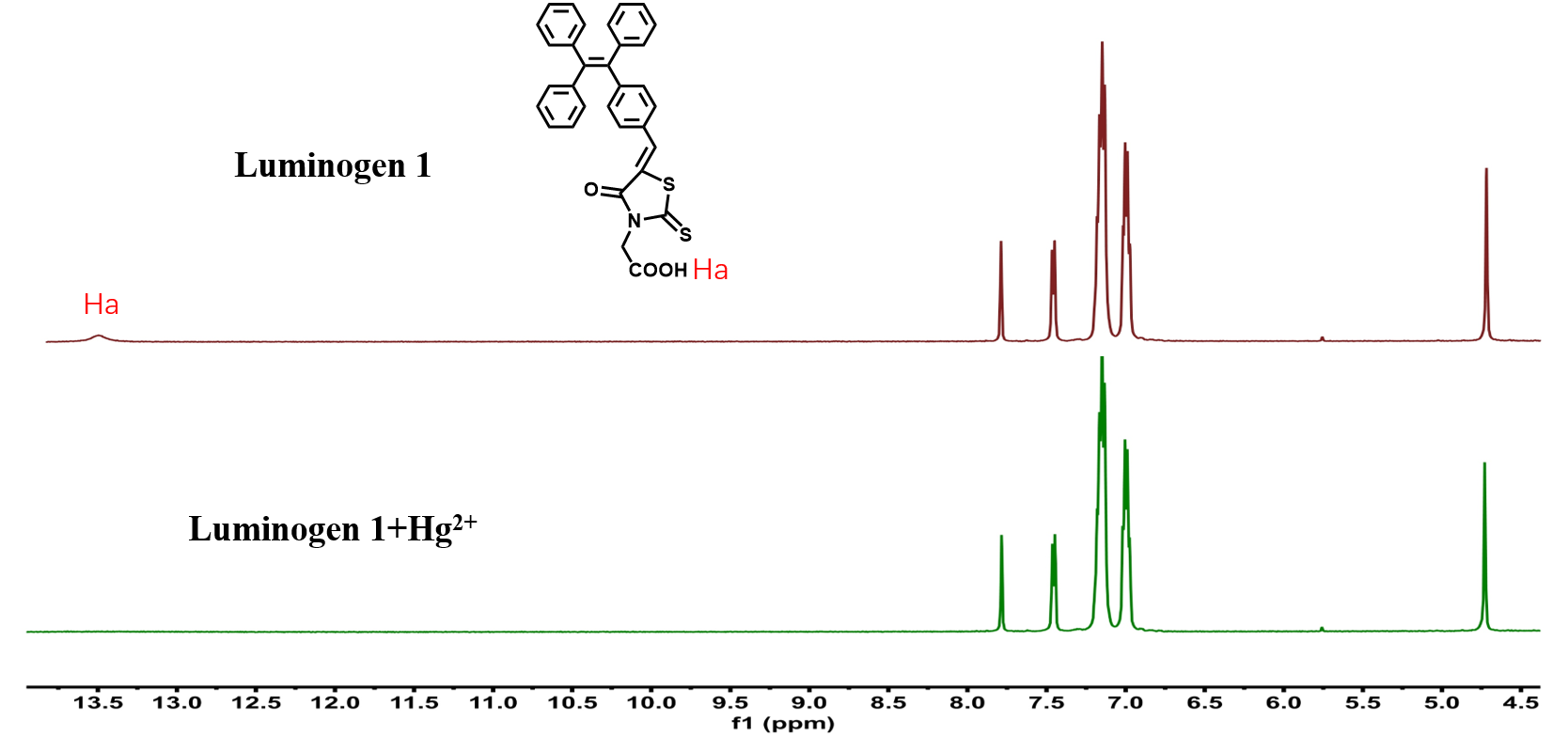


**(A)**


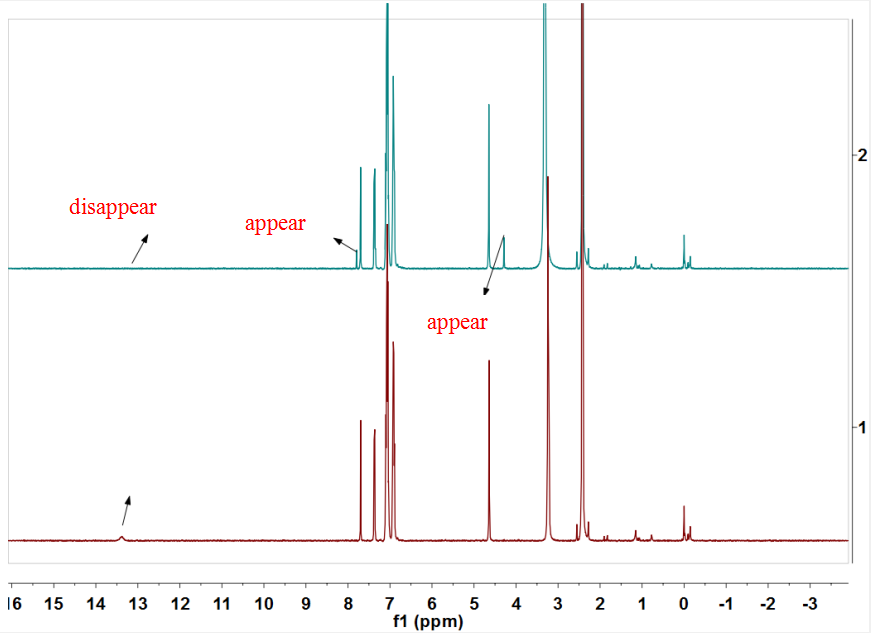


**(B)**

**FIGURE S8 | (A)** ^1^H NMR (dimethyl sulfoxide-d_6_, 500 HMz) spectra changes of compound **1** in the presence of Hg^2+^. **(B)** ^1^H NMR (dimethyl sulfoxide-d_6_, 500 HMz)

spectra changes of compound **1** in the presence of Hg^2+^. (Hg^2+^ solution prepared with DMSOd)


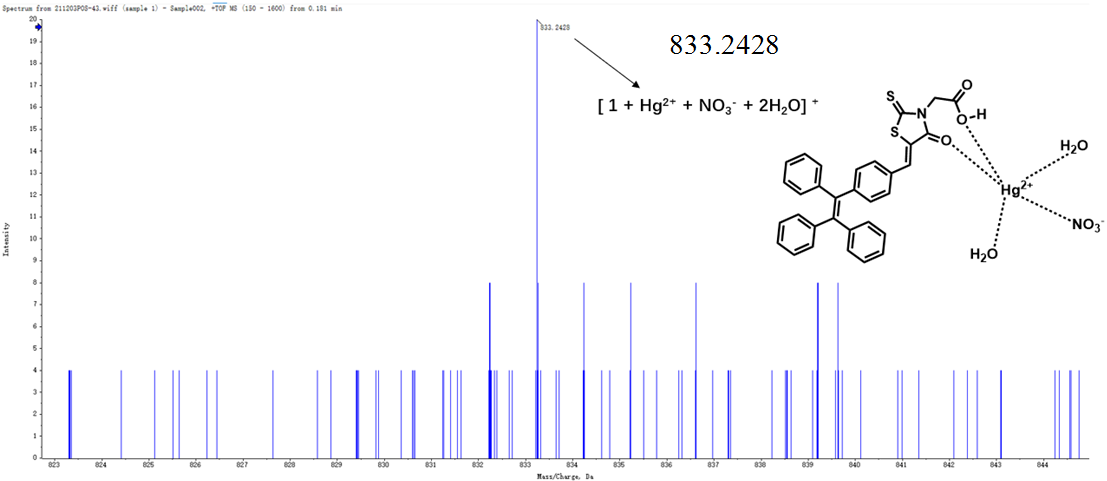


**FIGURE S9 |** Mass spectrum of compound **1** with Hg^2+^.


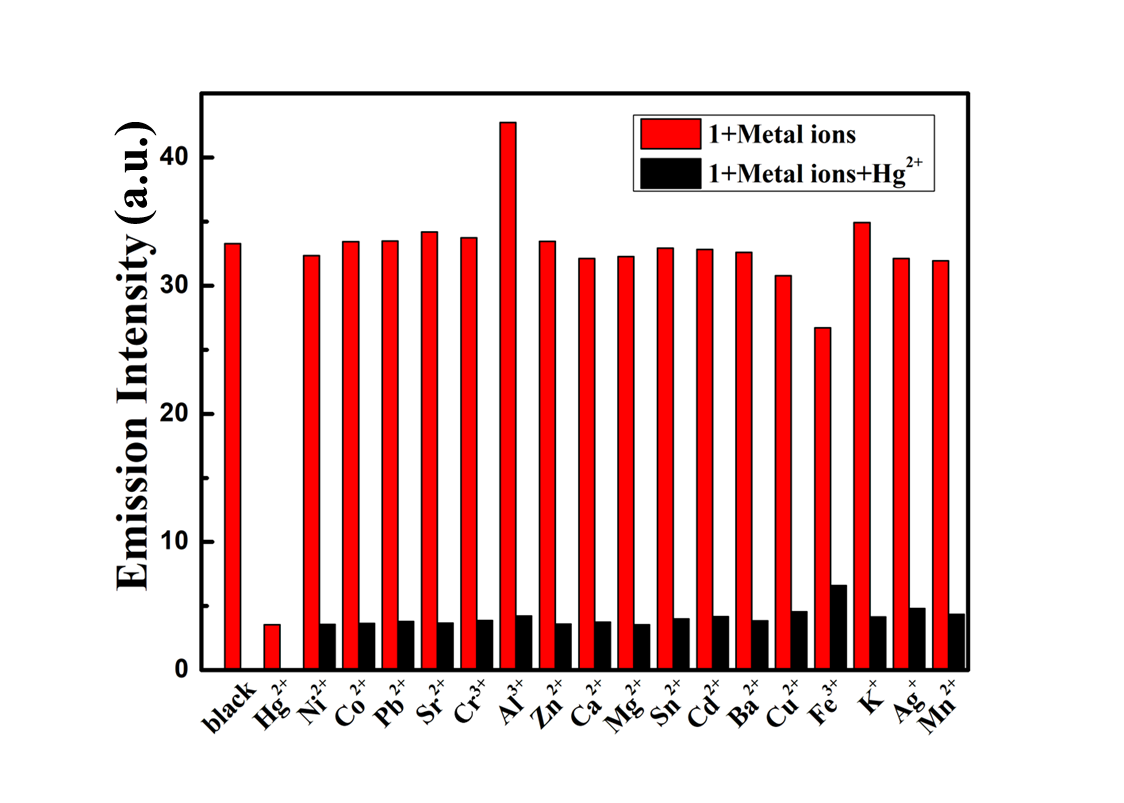


**FIGURE S10 |** Competitive tests of chemosensor **1** (concentration: 2.0 × 10^-5^ mol L^-1^) in the presence of various metal ions (10.0 equiv.) and subsequent addition of Hg^2+^ (10.0 equiv.).


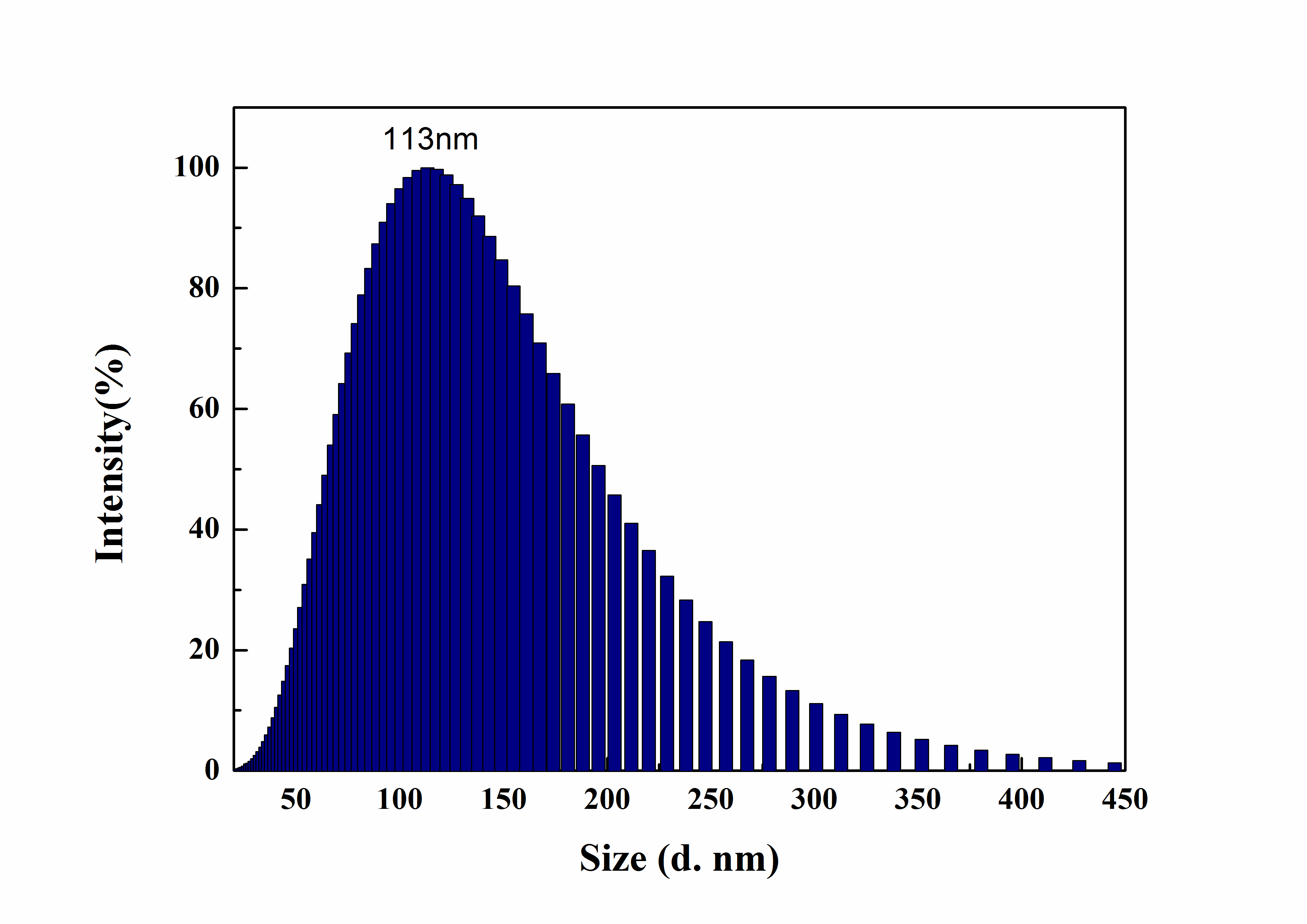


**FIGURE S11 |** Size distribution curve of compound **1** (2.0 × 10^-5^ mol L^-1^) in CH_3_CN-H_2_O mixture with 70% water fraction.


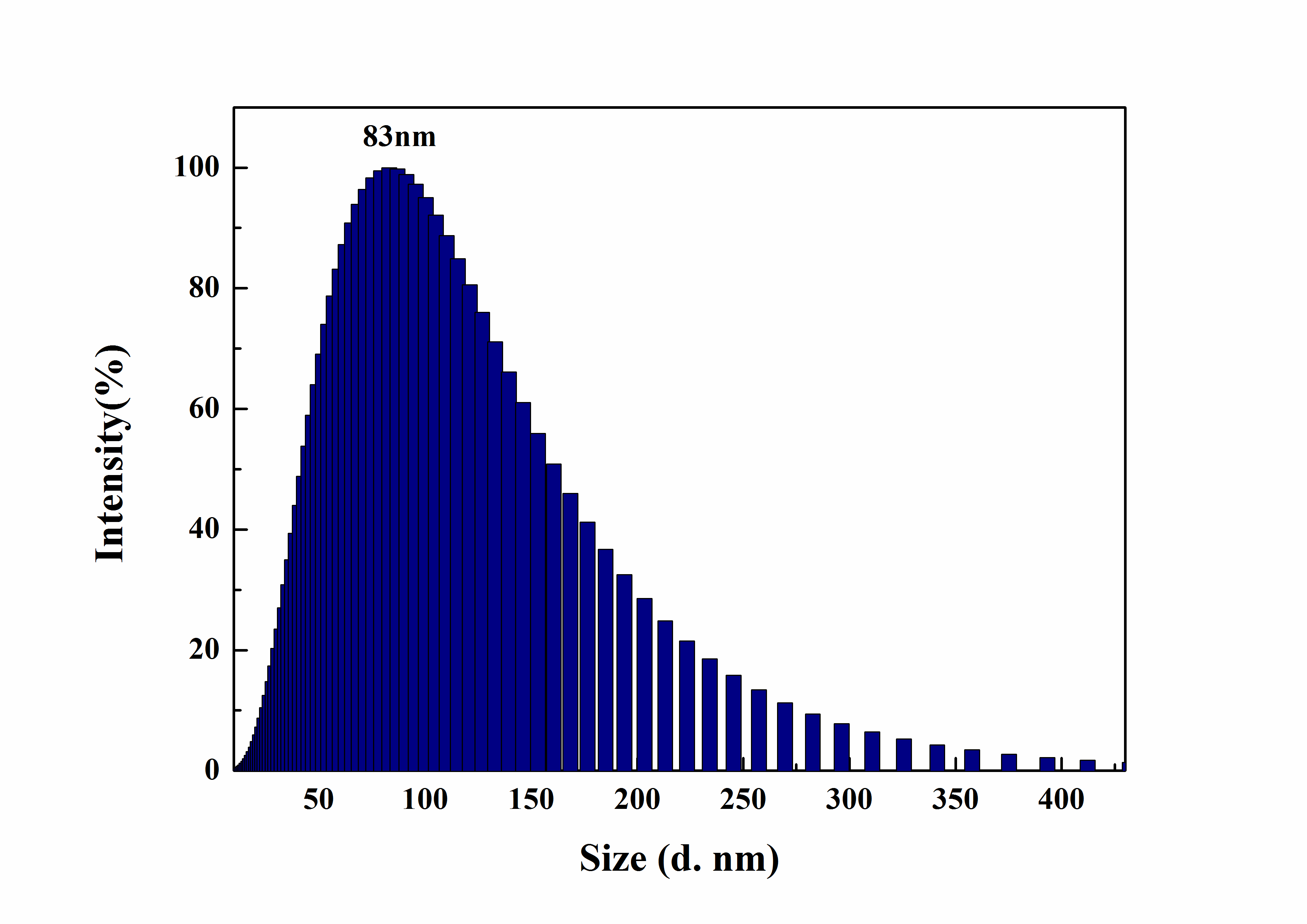


**FIGURE S12 |** Size distribution curve of compound **1** (2.0 × 10^-5^ mol L^-1^) in CH_3_CN-H_2_O mixture with 80% water fraction.


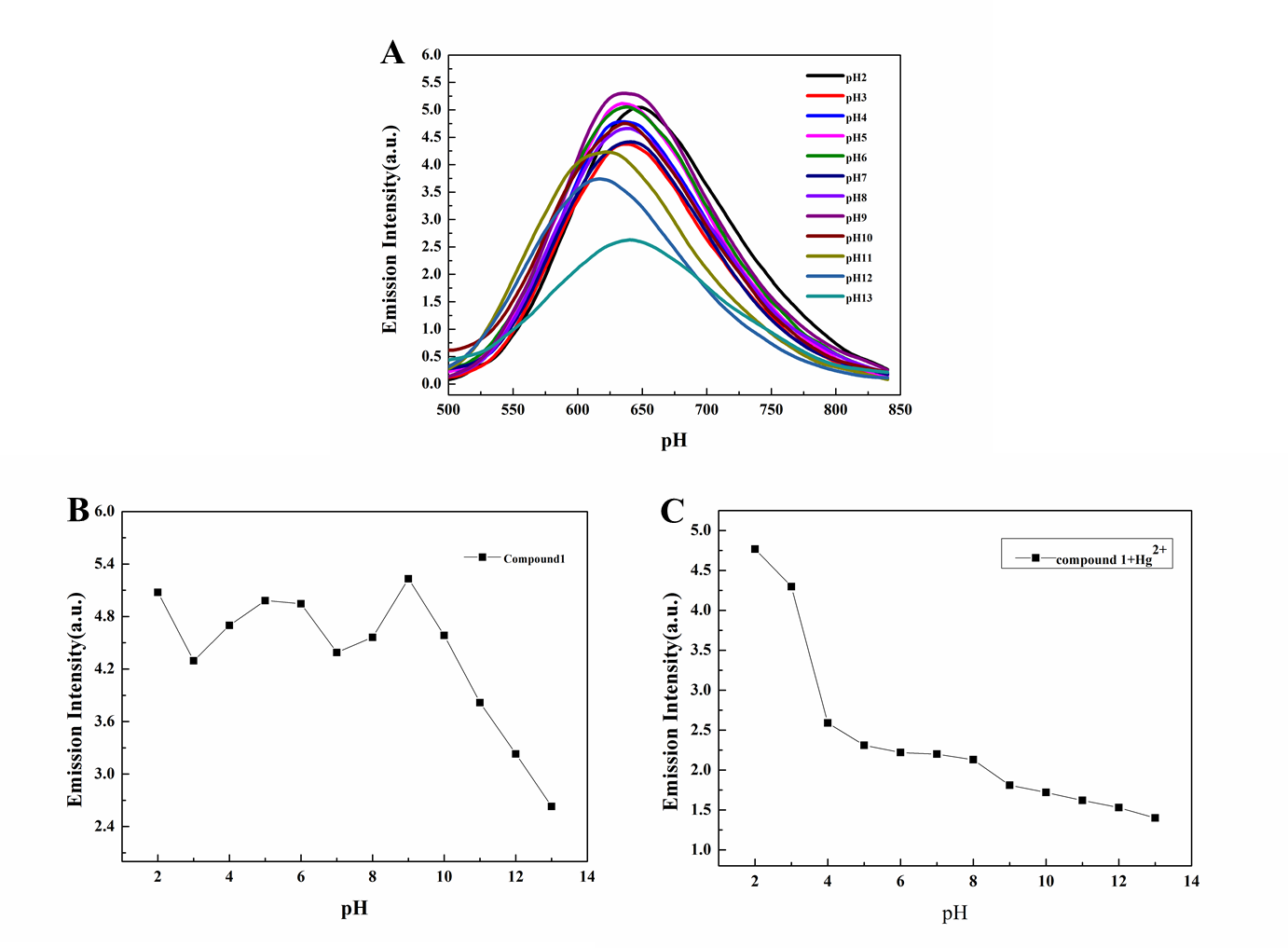


**FIGURE S13 |** **(A)** Fluorescence emission spectra of compound **1**(2.0 × 10^-5^ mol L^-1^) in the range of pH 2-13. **(B)** The change curve of fluorescence intensity and pH of compound **1** at 649nm. **(C)** The change curve of fluorescence intensity and pH of compound **1** with Hg^2+^ at 649 nm. Excitation wavelength = 425 nm Excitation slit =2.5 Emission slit = 2.5.

1. **Copies of NMR spectra and Mass spectrum**

^1^H NMR spectrum of compound **1**

^13^C NMR spectrum of compound **1**

Mass spectrum of compound **1**
